# The effect of azelaic acid on AlCl_3_-induced neurocognitive impairments and molecular changes in the hippocampus of rats

**DOI:** 10.1017/neu.2024.55

**Published:** 2024-02-17

**Authors:** Saba Vasegh, Hakimeh Saadati, Ali Abedi, Sara Mostafalou

**Affiliations:** 1 Department of Pharmacology & Toxicology, School of Pharmacy, Ardabil University of Medical Sciences, Ardabil, Iran; 2 Department of Physiology, School of Medicine, Ardabil University of Medical Sciences, Ardabil, Iran

**Keywords:** Cognitive impairment, azelaic acid, oxidative stress, neuroinflammation, aluminium chloride

## Abstract

**Objectives:**

Cognitive function plays a pivotal role in assessing an individual’s quality of life. This research aimed to investigate how azelaic acid (AzA), a natural dicarboxylic acid with antioxidant and anti-inflammatory properties, affects aluminium chloride (AlCl_3_)-induced behavioural changes and biochemical alterations in the hippocampus of rats.

**Methods:**

Thirty-two male Wistar rats divided into four groups received distilled water, AzA 50 mg/kg, AlCl_3_ 100 mg/kg and AzA plus AlCl_3_, respectively, by oral gavage for 6 weeks. Behavioural changes were evaluated using open-field maze, elevated plus maze, novel object recognition (NOR), passive avoidance task, and Morris water maze (MWM) tests. Also, malondialdehyde (MDA), carbonyl protein, tumour necrosis factor-alpha (TNF-α), interleukin-1 beta (IL-1β), nuclear factor-kappa B (NF-κB), C/EBP homologous protein (CHOP), glycogen synthase kinase-3 beta (GSK-3β), brain-derived neurotrophic factor (BDNF) and acetylcholinesterase (AChE) activity were examined.

**Results:**

AzA significantly affected AlCl_3_-provoked anxiety-like behaviours and learning and memory impairments. It also reduced the toxic effect of AlCl_3_ on MDA, carbonyl protein, TNF-α, IL-1β, NF-κB and GSK-3β status; however, its beneficial effects on AlCl_3_-induced changes of CHOP, BDNF and AChE activity were not significant.

**Conclusion:**

These findings disclosed that AzA could improve behavioural and cognitive function and almost limit the oxidative stress and neuroinflammation caused by AlCl_3_.

## Significant outcomes

- Sub-chronic administration of azelaic acid mitigated anxiety-like behaviours and ameliorated learning and memory deficits induced by sub-chronic exposure to aluminium chloride in Wistar rats.
- Sub-chronic administration of azelaic acid ameliorated inflammation, oxidative stress and glycogen synthase kinase-3 beta in the hippocampus of Wistar rats after sub-chronic exposure to aluminium chloride.

## Limitations

Despite extensive efforts to maintain cleanliness, silence and control of ventilation and light in the laboratory for experimental animals in accordance with standards, it was possible for these conditions to not be fully implemented throughout the entire procedure of the study which could potentially affect the behavioural changes of the animals.

## Introduction

Cognitive performance is processed by the interaction between neural networks. As the most complicated brain function, it consists of executive function, social cognition, attention, learning, memory, language and verbal fluency, and perceptual-motor function (Harvey, 2019, Butterfield and Boyd-Kimball, 2019).

Dysfunction of cognitive performance is not described only as memory impairments but a decline in a range of cognitive abilities or general cognition. As outlined in the fifth edition of the Diagnostic and Statistical Manual of Mental Disorders (DSM-5), neurocognitive disorders encompass delirium, mild neurocognitive disorder, and major neurocognitive disorder. Major neurocognitive disorder (previously categorised as dementia) is a severe condition in which the individual loses the ability to perform everyday activities independently. Mild cognitive impairment includes a noticeable change in cognition that does not interfere with the capability of independence in everyday activities, but it may progress to dementia or may not (Livingston *et al*., 2020).

Cognitive deficits arise from various factors, including ageing and psychiatric disorders like schizophrenia, bipolar disorder, and major depressive disorder. Additionally, neurodegenerative diseases such as Alzheimer’s and Parkinson’s can result in cognitive decline due to alterations in neuroanatomical structures like the hippocampus and prefrontal cortex (McDonald, 2017).

Oxidative stress and neuroinflammation are components of neurodegeneration and cognitive decline. Even though, they have two different pathologies, oxidative stress and neuroinflammation are related and can stimulate one another. Oxidative stress refers to the imbalance of oxidation-reduction and failure of antioxidant system, leading to the aggregation of reactive species, particularly reactive oxygen species (ROS) and reactive nitrogen species (RNS). Although the reactive species are necessary for physiological functioning, their overproduction can cause macromolecular damage, resulting in disruption of signalling mechanisms and cell death. The inflammatory response of the central nervous system to neurological events is termed as neuroinflammation, which is a protective response at the beginning but can lead to the degeneration of neurones. Actually, neuroinflammatory cells produce reactive species, causing oxidative stress and, in turn, some reactive species induce the expression of pro-inflammatory genes (Teleanu *et al*., 2022).

Heavy metals are remarkable neurodegeneration-provocative agents. Aluminium (Al) is one of the most distributed metals in the Earth’s crust, which can be significantly neurotoxic. Although it has no physiological role in the body, the human body is widely exposed to Al compounds due to its extensive use in industry, medical and pharmaceutical products, antiperspirants, food additives, agriculture, and water treatment (Igbokwe *et al*., 2019). Al can develop changes in blood-brain barrier (BBB), resulting in its accumulation in the brain (Wang, 2018). The neurodegenerative effect of Al is mainly utilised by stimulation of iron compounds to produce ROS, which leads to oxidative stress and inflammatory responses of the brain, leading to neuroinflammation. Also, Al can cause a disturbance in cholinergic function and neurotransmission, which is connected to cognition and behavioural responses (Colomina and Peris-Sampedro, 2017).

Azelaic acid (AzA) is a saturated dicarboxylic acid whit chemical formula of HOOC(CH_2_)_7_COOH found naturally in wheat, rye and barley (PubChem, 2004). This organic compound is used as a topical agent for treating mild to moderate acne. It has several biological activities, including anti-inflammatory and anti-oxidative effects (Sauer *et al*., 2023) and showed remarkable neuroprotective effects particularly against Parkinson’s disease (Sharmaa *et al*., 2021, Gan *et al*., 2011). A study on mice models of psoriasis showed that AzA could inhibit phosphatidylinositol 3-kinase (PI3K)/protein kinase B (Akt)/mammalian target of rapamycin (mTOR) signalling pathway which is involved in cell death processes (Li *et al*., 2022). Despite previous findings, AzA has never been studied for its potential protection against cognitive dysfunction. Thus, the current study evaluated the effect of AzA on aluminium chloride (AlCl_3_)-induced behavioural changes and cognitive disorder in association with biochemical and molecular alterations in the hippocampus of rats.

## Materials and methods

### Chemicals

ELISA kits for measuring activity of GSK-3β and BDNF were obtained from Millipore Corporation and Hangzhou Eastbiopharm Co., respectively. Tripure Isolation Reagent and Expand Reverse Transcriptase were purchased from Roche Applied Sciences. The primers were synthesised and delivered by GenFanavaran Ldt. For real-time PCR reaction, we used SYBR®Premix Ex Taq from Takara Bio Inc. AlCl_3_, AzA, acetylthiocholine, 5,5’-dithiobis-2-nitrobenzoic acid (DTNB), thiobarbituric acid, dinitrophenylhydrazine and other chemicals were purchased from the Sigma Aldrich Co.

### Experimental animals

In this study, a total of 32 male Wistar rats aged 3 months and weighing 200 – 250g were obtained from the Pasteur Institute of Iran. The animals were accommodated under standard laboratory conditions in standard cages (45.5 × 26.6 × 18 cm) (4 rats per cage) with 23 ± 1°C temperature and 12h light/dark cycle. All rats had free access to food and water and housed in a same room throughout the study. All the experiments were performed between 8:00 and 14:00 during the light cycle.

### Experimental design

All rats were divided into four groups of eight each, and treated with chemicals by oral gavage for six weeks. The first group (control group) received distilled water (1 ml). The second group received 50 mg/kg/day AzA dissolved in distilled water. The third group was administered 100 mg/kg AlCl_3_ suspended in distilled water. The fourth group was co-treated with the same dose of AzA and AlCl_3_ as used in the second and third groups. The doses of AlCl_3_ and AzA used in this study were selected based on previous research demonstrating their respective neurotoxic and neuroprotective effects. The dose of 100 mg/kg of AlCl3 has been widely used in animal models to induce neurotoxicity and cognitive impairments, replicating conditions similar to those seen in neurodegenerative diseases like Alzheimer’s (Zhang *et al*., 2022). The 50 mg/kg dose of AzA was chosen based on its efficacy in previous studies where it exhibited significant neuroprotective effects without causing adverse reactions (Sharmaa *et al*., 2021). The dual administration protocol, with a 2-hour interval between the gavage of AzA and AlCl_3_ in the AzA+AlCl_3_ group, was implemented to minimise potential interactions in the gastrointestinal tract, thereby ensuring the bioavailability and effectiveness of both compounds.

### Behavioural tests

All behavioural examinations were performed in a noise and light-controlled room between 8 am to 1 p.m. Animals were given 1 h to adapt the testing environment before each test. A blinded observer recorded all the tests. Each testing apparatus was cleaned with 70% ethanol prior to the placement of each rat. Behavioural tests were conducted according to the following schedule:

- Day 1: Open field test (also served as the habituation session for the novel object recognition test).
- Day 2: Novel object learning session.
- Day 3: Novel object testing session and elevated plus maze (EPM) test.
- Day 4: Morris Water Maze (MWM) test for the first half of the rats.
- Day 5: MWM test for the second half of the rats, and shuttle box habituation and learning session for the first half of the rats.
- Day 6: Shuttle box testing session followed by anaesthesia and hippocampus extraction on the first half of the rats, shuttle box habituation and learning session for the second half of the rats.
- Day 7: Shuttle box testing session followed by anaesthesia and hippocampus extraction on the second half of the rats.

#### Open-field maze

Open-field maze (OFM) is widely used for testing animals’ anxiety-like behaviours. The test was carried out on a wooden box with dimensions 60 × 60 × 50 cm, black walls and a white floor divided by black lines to 16 squares. At the start of the test, each animal was being placed in the central zone of the chamber for a period of five min, and the level of anxiety were evaluated by variables including total time of activity, time spent in inner zone, number of entries to the inner zone and the frequency of rearing and grooming (Sadegzadeh *et al*., 2020).

#### Elevated plus maze

The EPM is also one of the mostly conducted tests for assessment of anxiety-like behaviours. The apparatus was made up of wood with four ‘+’ shaped arms and a central zone, placed about 50 cm above the floor. Two of the opposite arranged arms were enclosed with walls (50 × 10 × 40 cm) and an open top. The other pair of arms were open (50 × 10 cm) and equipped with 0.5 × 0.5 cm ledge to avoid rats from falling down. The test begins by placing an animal in the central zone, facing one of the open arms, and lasts for up to 5 minutes. Recorded factors include the time spent in the open arms (*T*_1_), time spent in the closed arms (*T*_2_), the frequency of entries into the open arms (*F*_1_), and the frequency of entries into the closed arms (F_2_). Anxiety levels are assessed by calculating the percentage of time spent in the open arms [T_1_/(T_1_+T_2_) × 100] and the percentage of entries into the open arms [F1/(F_1_+F_2_) × 100]. Additionally, the total number of entries into the arms serves as an indicator of locomotor activity (Sadegzadeh *et al*., 2020).

#### Novel object recognition test

The novel object recognition (NOR) test is one of the most comparable recognition tests to those used in humans (Brodziak *et al*., 2014). The test is completely based on rodents’ natural curiosity for novel objects and the ability to recognise them without any primary reinforcement. The protocol of the test consists of three sessions: habituation, training and testing. During the habituation, each animal was placed in the open-field chamber to explore the environment without any objects in it for five min. After a 30-min interval, the training session was performed by placing the animal in the open-field box containing two identical objects for 10 min. After 24 hour, each rat was returned to the chamber and exposed to a familiar object and a novel one. The novel object was completely distinguishable for the animals, even though all of them were made up of the same material and were heavy enough for animals to displace. Each animal was allowed to explore the chamber for five min. The total time spent on investigating the novel object (*T*_1_) and the total time of interaction with either object (*T*_t_) in testing phase were calculated to determine the discrimination ratio (100×T_1_/T_t_) (Sadegzadeh *et al*., 2020).

#### Morris water maze

The spatial and short-term learning and memory of the animals were assayed by the Morris water maze (MWM) test. The test occurred in a black pool with 160 cm diameter, 80 cm height and 40 cm depth. The pool was equally separated into four quadrants (no.1– no.4). A rounded platform with 10 cm diameter was located in the second quadrant 1.5 cm below the water surface. The platform was black in order to be indistinguishable from the environment. Visual cues were fixed on the walls around the pool. A video camera connected to a computerised tracking system (Noldus Ethovision® system, version 5, USA) was set above the centre of the pool to record each session.

The MWM task was accomplished in two sessions of learning and testing. In the spatial learning phase, each animal was released into the pool from the 1^st^, 3^rd^ and 4^th^ quadrants and allowed to detect the platform during a 60s- interval in four training trials. Rats was given 60 second-rest between the trials. Each trial was repeated in three blocks with a 30-min interval between them. After detection of the platform, rats were permitted to rest on the platform for 30 s in order to analyse the visual signals before being put back in its cage. Each rat was dried with a towel right after returning to the cage and was allowed to rest for 30 s before the next trial. However, if a rat could not discover the platform within 60 s, the experimenter would put it on the platform. In this phase, the tracking system recorded the two parameters of latency and the distance travelled to find the platform. At the end, the average of the parameters in each trial for every block was reported. The testing phase was performed two hours after the end of the learning phase. To evaluate the spatial memory, the platform was removed from the pool and each rat was released into the water with the same order as in the previous phase. The time spent and the distance moved in the target quadrant and the number of crossings were used to measure spatial memory retention (Saadati *et al*., 2015).

#### Passive avoidance task

Passive avoidance is a fear-motivated learning and memory test in which the animal learns NOT to enter an environment to avoid the punishment (electric foot shock) that was previously experienced, despite its natural photophobia. The test starts with two min of habituation in the light compartment (LC) of the light/dark chamber. After two min, each rat was put back in the LC for the training phase. In this phase the animals spent 20 s in the LC before opening the guillotine door between the two compartments. As soon as the rat entered into the dark compartment (DC) the door was closed and a 1.5 mA of foot shock was applied for 2 s. The testing trial was carried out 24 h after training. Each rat was placed in the LC of the apparatus in the same manner as the training session. The door was opened after a 20 s period and the animal was allowed to explore the apparatus for 300 s. During the testing trial, step through latency (STL), frequency of entries into the DC and total time spent in DC were measured as the index of learning and memory (Golitabari *et al*., 2022).

### Biochemical analyses

Following behavioural testing, rats were euthanized by decapitation under light anaesthesia, and their brains were rapidly removed and the hippocampus was dissected and homogenised for subsequent biochemical analyses.

#### Quantification of oxidative stress markers in the hippocampus

To detect the dimensions of oxidative stress, we measured the markers of lipid peroxidation and oxidative damage to proteins. Thiobarbitoric acid reactive substances (TBARS) were measured spectrophotometrically as the indicator of lipid peroxidation products such as malondialdehyde (MDA). Results were expressed as mg/g tissue as ε =153 M^−1^cm^−1^. The oxidative damage to proteins was evaluated by quantification of carbonyl content based on the reaction with dinitrophenyl hydrazine. The carbonyl content (nmol/mg protein) was assessed using a molar extinction coefficient of 22,000 M^−1^cm^−1^ at 370 nm after subtraction of the blank absorbance (Mostafalou *et al*., 2015).

#### Investigation of gene expression changes by real-time PCR in the hippocampus

To assess changes in gene expression of nuclear factor-kappa B (NF-κB), interleukin-1 beta (IL-1β), tumour necrosis factor-alpha (TNF-α) and C/EBP houmolougous protein (CHOP), real-time PCR was conducted following mRNA extraction and cDNA synthesis (Mousavi-Nasab *et al*., 2024). RNA was extracted from sample tissue solution using the BioFACT kit, and its concentration was determined using a Nano-Drop UV–vis Spectrophotometer (Thermo Fisher Scientific, CA). Subsequently, the RNA was reverse transcribed into cDNA using the BioFACT cDNA synthesis kit, with a concentration of 1 μg/μl RNA. Specific primers were designed and obtained from GenFanavaran Ldt. The real-time PCR reaction was performed using the SYBR green master mix. Cycle number (Ct) of each reaction was achieved from Light Cycler 96 (Roche Applied Sciences, USA). The values were normalised to GAPDH mRNA and the relative gene expression level was represented as 2^−ΔΔCt^. The following primers were used in this study: CHOP forward: CGGAGTGTACCCAGCACCATCA, CHOP reverse: CCCTCTCCTTTGGTCTACCCTCA; NF-kB forward: TTCAACATGGCAGACGACGA, NF-kB reverse: AGGTATGGGCCATCTGTTGA; IL-1β forward: AGCCAGAGTCATTCAGAGCAA, IL-1β reverse: GTCCTTAGCCACTCCTTCTG; TNF-α forward: ACACACGAGACGCTGAAGTA, TNF-α reverse: TCCACTCAGGCATCGACATT; GAPDH forward: GTATGACTCTACCCACGGCA, GAPDH reverse: AAGACGCCAGTAGACTCCAC.

#### Measurement of acetylcholinesterase activity in the hippocampus

A colorimetric kinetic method was used to determine acetylcholinesterase (AChE) activity in the hippocampus. A 96-well microtiter plate was filled with the samples, acetylthiocholine (Ach) as the substrate, and 5,5’-dithiobis-2-nitrobenzoic acid (DTNB) as the colorimetric reagent. The thiocholine, produced from the hydrolysis of the substrate by AChE, reacted with DTNB to yield a yellow-colored anion, 5-thio-2-nitrobenzoate (TNB). The rate of TNB formation was kinetically measured using an automated microtiter plate reader at 412 nm (Alivand *et al*., 2024, Mahdavi *et al*., 2024).

#### Measurement of glycogen synthase kinase-3beta (GSK-3β) activity in the hippocampus

The activity of this serine/threonine kinase was assessed by using the STAR (Signal Transduction Assay Reaction) phospho-GSK-3 (Ser9) ELISA Kit, Catalog number 17-472, Millipore Corporation.

#### Measurement of brain-derived neurotrophic factor (BDNF) level in the hippocampus

The hippocampus samples were homogenated in lysis buffer and centrifuged at 12000g (at −4°C for 20 min). We used Rat BDNF ELISA Kit (Hangzhou Eastbiopharm Co., LTP) and followed the manufacturer instructions for assessing the brain derived neurotrophic factor (BDNF) protein level in the extracted supernatant.

### Statistical analysis

The results were presented as mean ± SEM. The normality of the data was assessed using the Shapiro-Wilk test. Statistical differences between groups were analysed using one-way ANOVA followed by LSD post-hoc test for behavioural tests and Tukey’s post-hoc for biochemical results. The *F*-test results for each ANOVA analysis were reported. The *p* value<0.05 was considered to be statistically significant. All statistical analyses were performed using SPSS version 25.0 (IBM Corp., USA).

## Results

### Open-field maze

**Figure 1.**
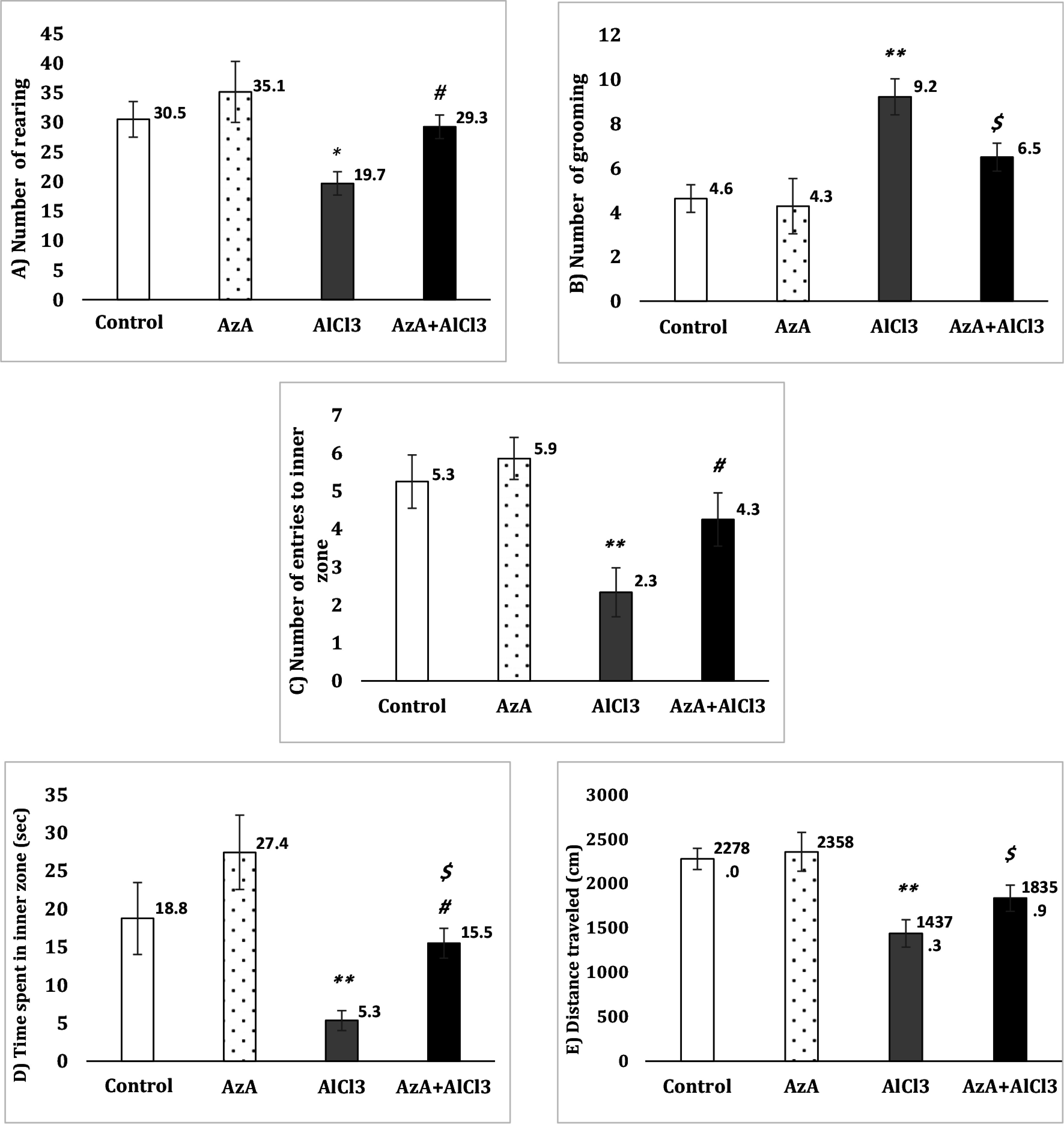
Effect of azelaic acid on the AlCl3-provoked anxiety-like behaviours in the open-field test. The results were represented as mean±SEM (one-way ANOVA). (*) *p* < 0.05 and (**) *p* < 0.01 compared with the control group, (#) *p* < 0.05 compared with the AlCl3 group and ($) *p* < 0.05 compared with the AzA group. AzA: azelaic acid.

As shown in the Fig. 1A, there was a significant decrease in the number of rearing in the AlCl_3_ group compared to the control group [F (3, 28) = 4.56, *p* < 0.05]. AzA significantly increased the number of rearing in the AzA+AlCl_3_ in comparison to the AlCl_3_ group (*p* < 0.05); however, when AzA group was compared to the control, the increment was not noticeable.

Also, the number of grooming was considerably increased in the AlCl_3_ group compared to the control group [*F* (3, 28) = 7.66, *p* < 0.01]. The number of grooming of the AzA+AlCl_3_ group was not significantly different than either the control group or the AlCl_3_ group. However, it was noticeably more than AzA group (*p* < 0.05). Though, AzA decreased the number of grooming in comparison to control group the change was not significant (Fig. 1B).

Analysis in the Fig. 1C shows a significant decrease in the number of entries to the inner zone when comparing the AlCl_3_ group to the control group [*F* (3, 28) = 5.63, *p* < 0.01]. Conversely, in the AzA+AlCl_3_ group, there was a notable reduction in the number of entries to the central area compared to the AlCl_3_ group (*p* < 0.05).

The Fig. 1D also shows that the AlCl_3_ caused a significant decrease in the time spent in the centre zone compared to the control group [*F* (3, 28) = 7.20, *p* < 0.01]. However, in the AzA+AlCl_3_ group, this time was significantly longer than that of the AlCl_3_ group (*p* < 0.05), although noticeably reduced compared to the AzA group (*p* < 0.05).

AlCl_3_ also led to a remarkable decrease in the distance travelled comparing with control [*F* (3, 28) = 7.34, *p* < 0.01]. However, co-treatment with AzA was not able to increase this parameter in comparison with AlCl_3_ group. The AzA+AlCl_3_ group also, showed a significant decrease in comparison to the AzA group (*p* < 0.05) (Fig. 1E).

### Elevated plus maze

**Figure 2.**
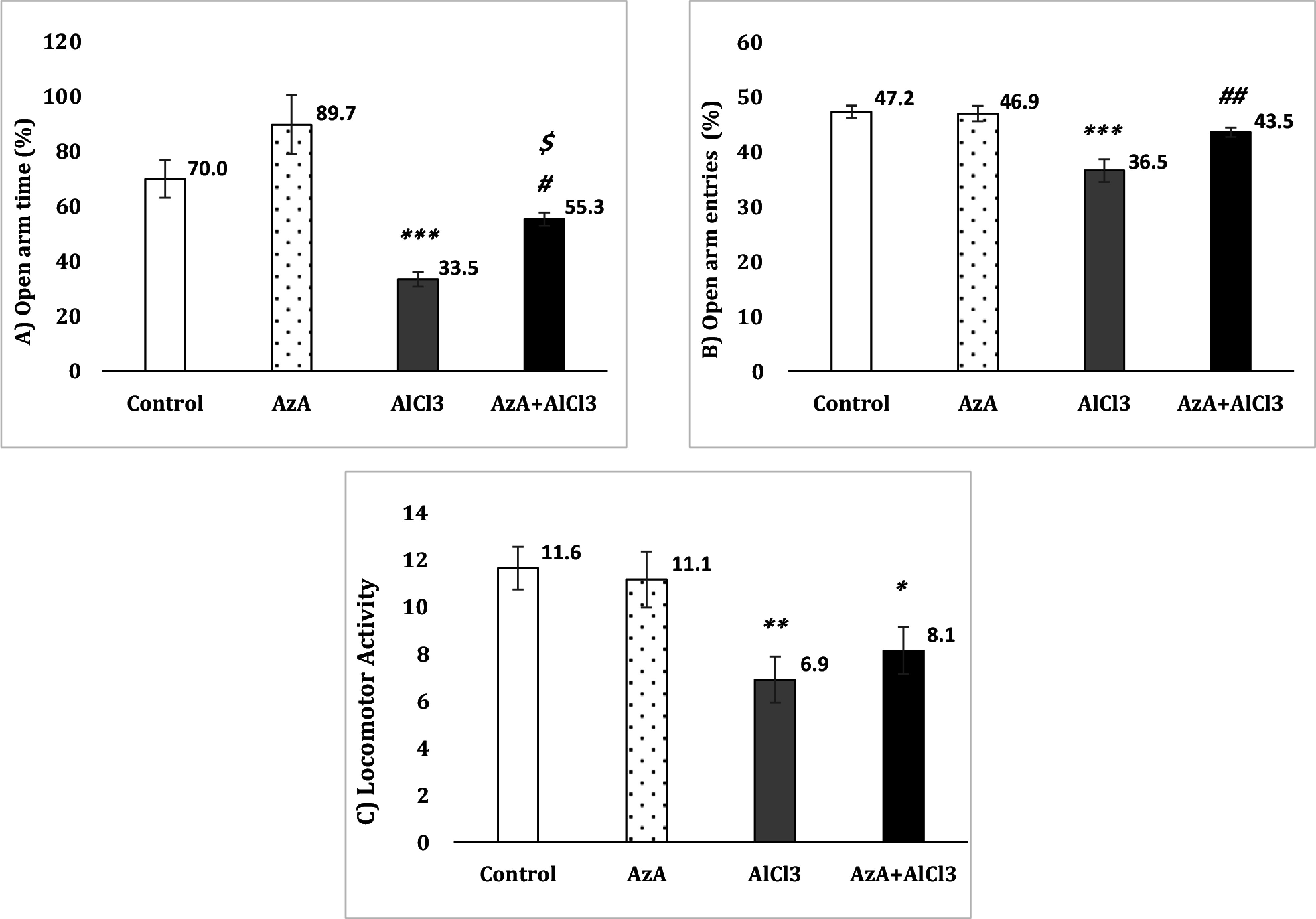
Effect of azelaic acid on the AlCl_3_-provoked anxiety-like behaviours in the elevated plus maze test. The results were represented as mean±SEM (one-way ANOVA). (*) *p* < 0.05, (**) *p* < 0.01 and (***) *p* < 0.001 compared with the control group, (#) *p* < 0.05 and (##) *p* < 0.01 compared with the AlCl_3_ group, ($) *p* < 0.05 compared with the AzA group. AzA: azelaic acid.

The Fig. 2A shows that the AlCl_3_ caused a considerable decrease in time spent in the open arm in comparison with the control group [*F* (3, 28) = 11.91, *p* < 0.001] while this time was significantly longer in the AzA+AlCl_3_ group compared to the AlCl_3_ group (*p* < 0.05) and the AzA group (*p* < 0.05). However, the AzA group indicated no remarkable change in comparison to the control group.

The number of entries to the open arm was considerably less in the AlCl_3_ group compared to the control group [*F* (3, 28) = 15.20, *p* < 0.001] whereas the AzA+AlCl_3_ group had significantly more open arm entries compared to the AlCl_3_ group following AzA intake (*p* < 0.01) (Fig. 2B).

Locomotor activity was notably decreased in the both AlCl_3_ [*F* (3, 28) = 5.33, *p* < 0.01] and AzA+AlCl_3_ in comparison to the control group, and there was no significant difference between the AlCl_3_ group and other three groups (Fig. 2C).

### Novel object recognition test

**Figure 3.**
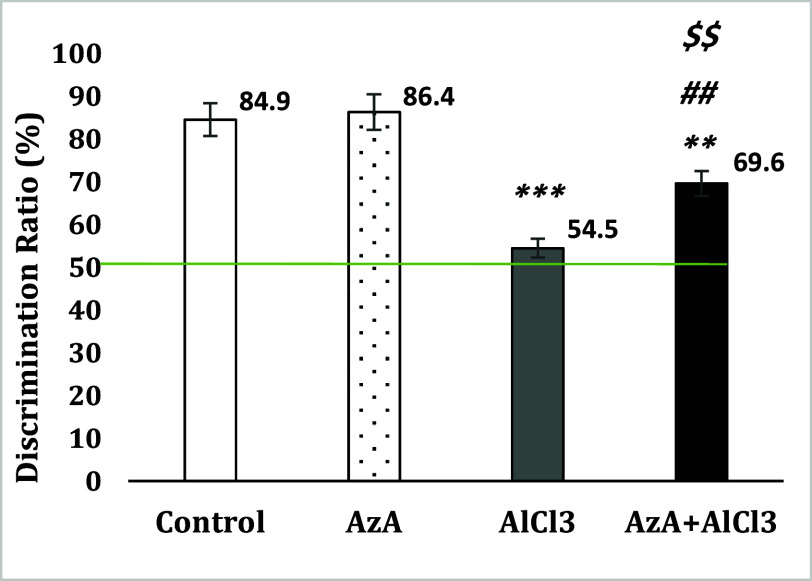
Effect of azelaic acid on the AlCl_3_-provoked learning and memory changes in the novel object recognition test. The results were represented as mean±SEM (one-way ANOVA). (**) *p* < 0.01 and (***) *p* < 0.001 compared with the control group, (##) *p* < 0.01 compared with the AlCl_3_ group and ($$) *p* < 0.01 compared with the AzA group. AzA: azelaic acid.

The results revealed that AlCl_3_ significantly decreased NOR in comparison to the control group [*F* (3, 28) = 21.74, *p* < 0.001] while AzA modified this cognitive disorder in the AzA+AlCl_3_ group and considerably improved the recognition in comparison to the AlCl_3_ group (*p* < 0.01). However co-treatment could not notably increase discrimination ratio compared to the control and AzA groups whose ratios were still significantly higher (*p* < 0.01) (Fig. 3).

### Morris water maze

**Figure 4.**
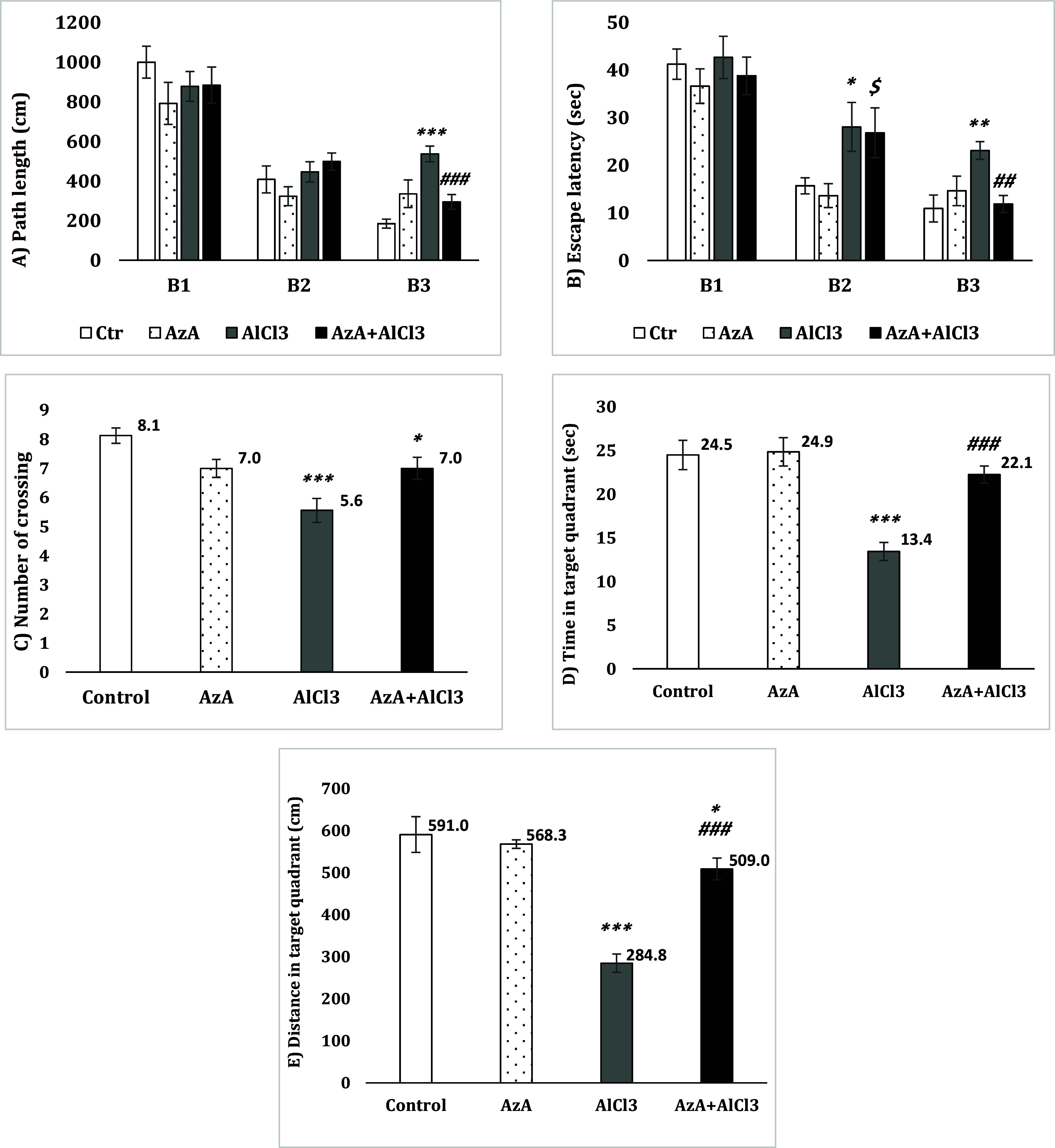
Effect of azelaic acid on the AlCl_3_-provoked memory impairment in the morris water maze test. The results were represented as mean±SEM (one-way ANOVA). (*) *p* < 0.05, (**) *p* < 0.01 and (***) *p* < 0.001 compared with the control group, (##) *p* < 0.01 and (###) *p* < 0.001 compared with the AlCl_3_ group, ($) *p* < 0.05) compared with the AzA group. AzA: azelaic acid, B1: block1, B2: block2, B3: block3.

In the learning session of the test, no noticeable difference was detected in the path length between the groups in both Block 1 and Block 2; however, in Block3, the AlCl_3_ group took a considerably longer path to reach the platform than the control group [F (3, 28) = 12.33, *p* < 0.001] but the AzA+AlCl_3_ group showed a significantly better performance compared to the AlCl_3_ group (p < 0.001) (Fig. 4A).

The time spent for finding the platform was analysed in each block. The data of block 1 detected no significant change between the groups. On the other hand, according to the data of block 2, the AlCl_3_ group showed a noticeably longer period of time than the control group [*F* (3, 28) = 3.17, *p* < 0.05]. The other groups represented no significantly different performance compared to the control group. But, the AzA+AlCl_3_ group showed significantly longer escape latency than the AzA group (*p* < 0.05). Compared to the control, the AlCl_3_ group also had a significantly increased time spent to find the platform in block 3 [*F* (3, 28) = 5.83, *p* < 0.01] while the AzA+AlCl_3_ group considerably spent less time than the AlCl_3_ group (*p* < 0.01) (Fig. 4B).

Three parameters were considered in the testing session: the time spent in target zone, number of crossing and the distance in target zone. The AlCl_3_ group crossed the target zone remarkably in less times than the control group [*F* (3, 28) = 9.13, *p* < 0.001]. The increase in the number of crossing of the AzA+AlCl_3_ group was not significant compared to the AlCl_3_ group and the crossing number of the AzA+AlCl_3_ group still was considerably less than the control group (*p* < 0.05) (Fig. 4C).

The time spent in target zone showed better results. The AlCl_3_ group spent significantly less time in the target zone compared to the control group [*F* (3, 28) = 17.05, *p* < 0.001], while there was a significant change between the AlCl_3_ and the AzA+AlCl_3_ groups (*p* < 0.001) (Fig. 4D).

The distance in target zone was considerably reduced in the AlCl_3_ group compared to the control group [*F* (3, 28) = 26.50, *p* < 0.001]. However, an absolute significant change were observed when comparing the AzA+AlCl_3_ and AlCl_3_ groups (*p* < 0.001). Yet, the AzA+AlCl_3_ group had moved less distance in the target zone than the control group (*p* < 0.05) (Fig. 4E).

### Passive avoidance task

**Figure 5.**
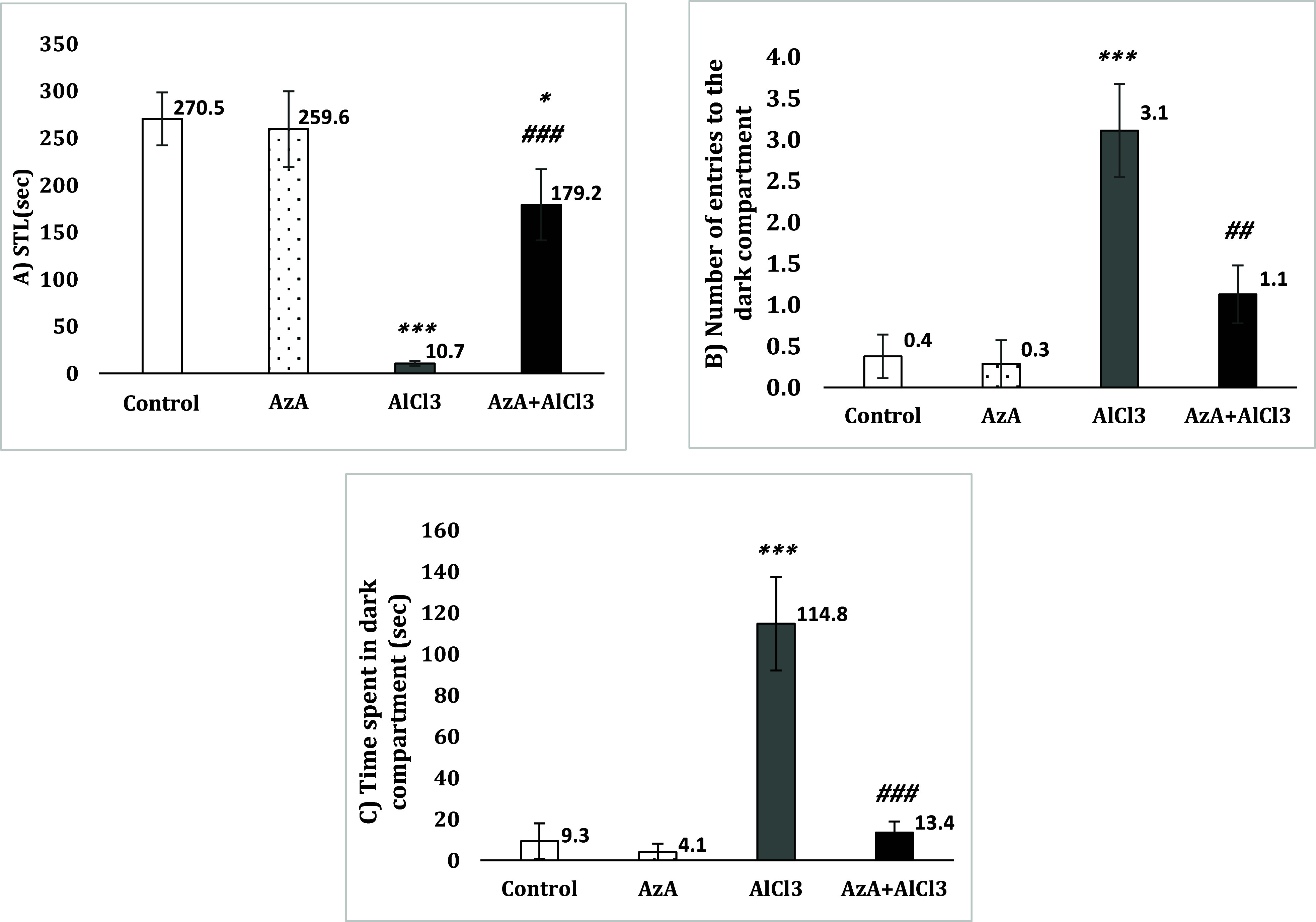
Effect of azelaic acid on the AlCl_3_-provoked behavioural changes in the passive avoidance learning and memory test. The results were represented as mean±SEM (one-way ANOVA). (*) *p* < 0.05 and (***) *p* < 0.001 compared with the control group, (##) *p* < 0.01 and (###) *p* < 0.001 compared with the alCl_3_ group. AzA: azelaic acid, STL: step through latency.

Analyses of STL indicated a remarkable decline of this parameter in the AlCl_3_ group compared to the control group [*F* (3, 28) = 17.65, *p* < 0.001]. Also, AzA noticeably increased STL in the AzA+AlCl_3_ group (*p* < 0.001). However this increase was not enough to raise STL to the level of the control group (*p* < 0.05) (Fig. 5A).

The number of entries to the DC did not show a significant change between AzA and control groups, while AlCl_3_ group entered the DC remarkably more often than the control group [*F* (3, 28) = 11.03, *p* < 0.001]. AzA+AlCl_3_ group had significantly less number of entries to this area than the AlCl_3_ group (*p* < 0.01), however (Fig. 5B).

Also, AlCl_3_ group spent considerably more time in the DC than the control and AzA groups [*F* (3, 28) = 15.56, *p* < 0.001]. The group AzA+AlCl_3_ also spent significantly less time in this area than the AlCl_3_ group (*p* < 0.001) (Fig. 5C).

### Oxidative stress markers in the hippocampus

**Figure 6.**
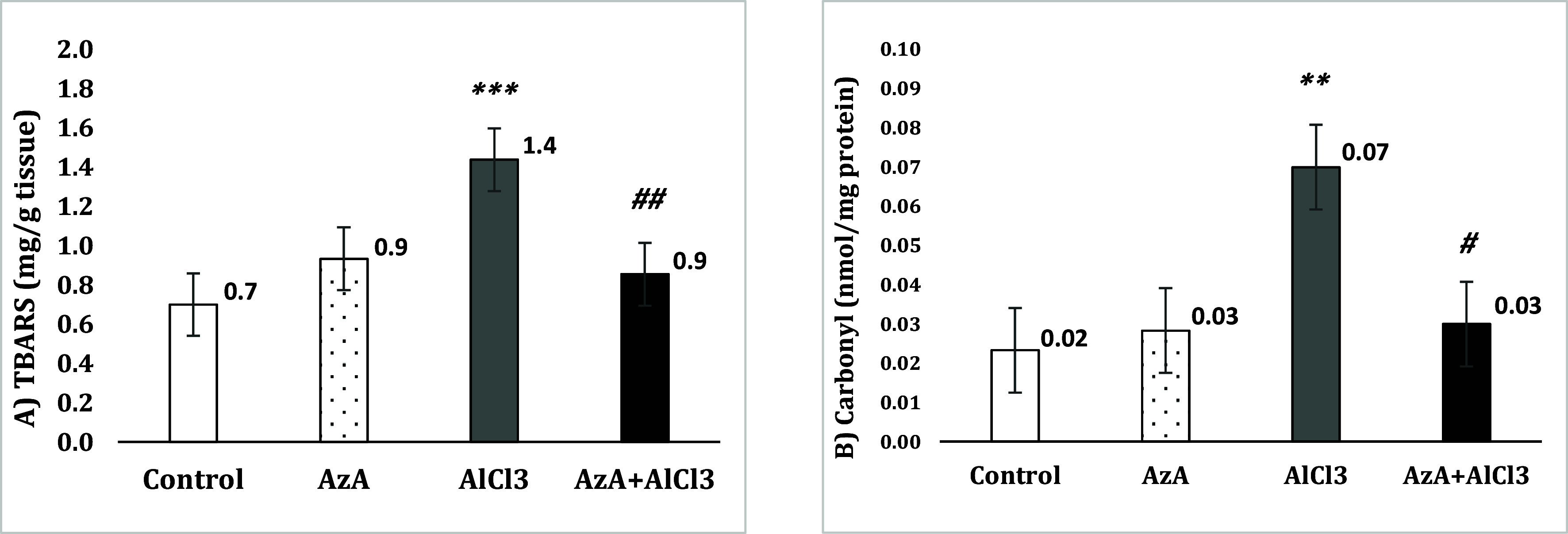
Effect of azelaic acid on AlCl_3_-induced oxidative stress in the hippocampus. The results were represented as mean±SEM (one-way ANOVA). (**) *p* < 0.01 and (***) *p* < 0.001 compared with the control group, (#) *p* < 0.05 and (##) *p* < 0.01 compared with the AlCl_3_ group. AzA: azelaic acid, TBARS: thiobarbituric acid reactive substances.

As shown in the Fig. 6A, significantly higher levels of TBARS was found in the AlCl_3_ group than the control group [*F* (3, 28) = 8.55, *p* < 0.001]. The AzA+AlCl_3_ group had significantly lower status of TBARS than the AlCl_3_ group (*p* < 0.05). However, there was no significant change between the control, AzA and AzA+AlCl_3_ groups.

There was a notable increase in carbonyl content in the AlCl_3_ group compared to the control group [*F* (3, 28) = 6.80, *p* < 0.01]. Notably, the AzA+AlCl_3_ group exhibited significantly lower carbonyl levels than the AlCl_3_ group (*p* < 0.05). However, no considerable change was observed between the AzA and control groups (Fig. 6B).

### Gene expression changes in the hippocampus

**Figure 7.**
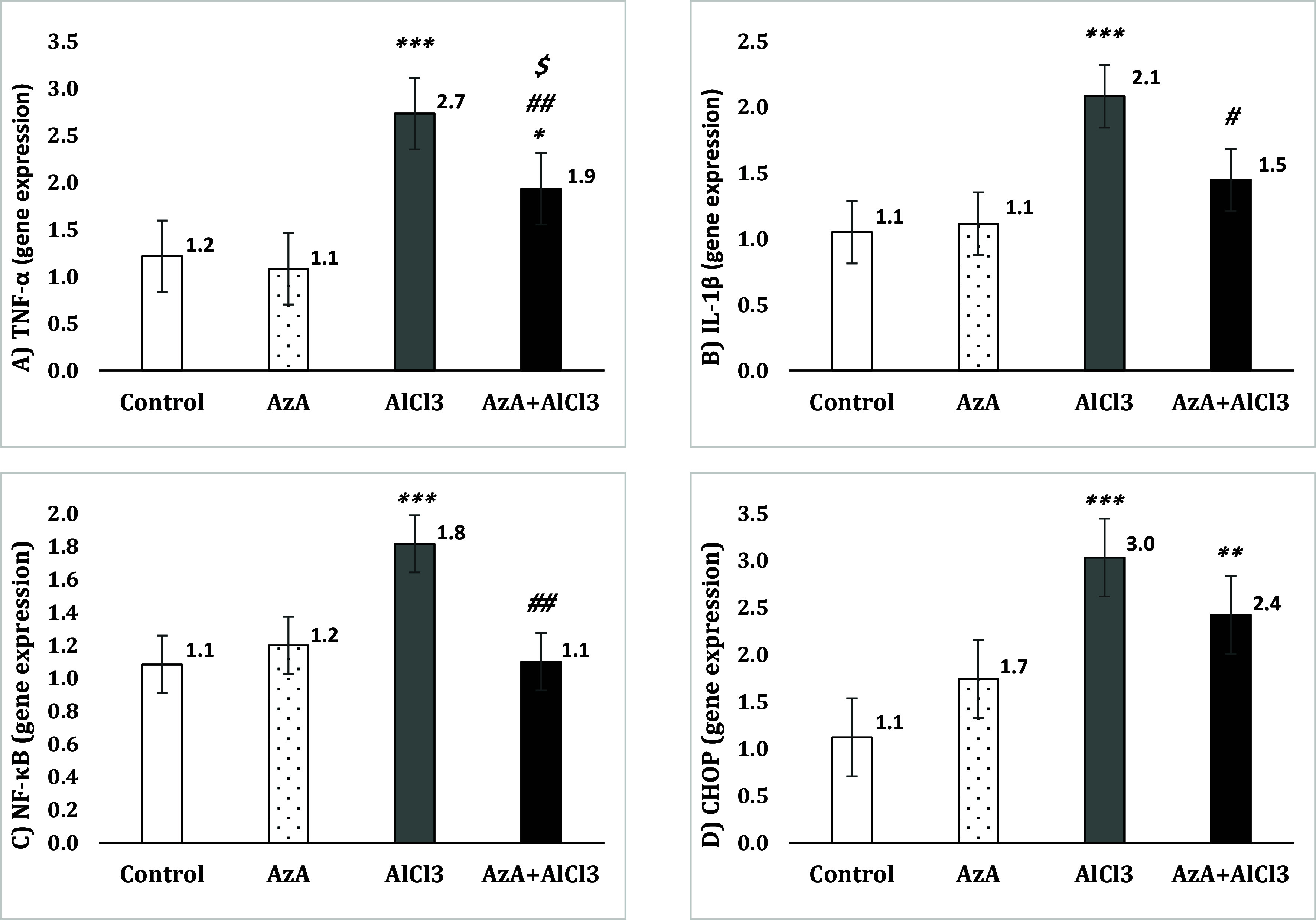
Effect of azelaic acid on the AlCl_3_-induced neuroinflammation in the hippocampus. The results were represented as mean±SEM (one-way ANOVA). (*) *p* < 0.05, (**) *p* < 0.01 and (***) *p* < 0.001 compared with the control group, (#) *p* < 0.05 and (##) *p* < 0.01 compared with the AlCl_3_ group and ($) *p* < 0.05 compared with the AzA group. AzA: azelaic acid, TNF-α: tumor necrosis factor-α, IL-1β: Interleukin-1β, NF-κB: nuclear factor kappa-light-chain-enhancer of activated B cells, CHOP: C/EBP homologous protein.

The evaluation of TNF-α gene expression revealed no remarkable change between the control group and the AzA group, while it was significantly increased in the AlCl_3_ group compared to the control group [*F* (3, 28) = 23.82, *p* < 0.001]. Although, the AzA+AlCl_3_ group was observed to have noticeably less levels of expression than the AlCl_3_ group (*p* < 0.01), it still showed significantly increased levels compared to the control group (*p* < 0.05) and the AzA group (*p* < 0.05) (Fig. 7A).

Though, the expression of IL-1β was not notably different in control, AzA, and AzA+AlCl_3_ groups, it was considerably increased in the AlCl_3_ group when compared to the control [*F* (3, 28) = 12.72, *p* < 0.001], AzA, and AzA+AlCl_3_ (*p* < 0.05) groups (Fig. 7B).

When compared to the control group, AlCl_3_ intake caused a remarkable promotion of NF-κB gene expression [*F* (3, 28) = 9.73, *p* < 0.001]; nevertheless, AzA significantly reduced it in AzA+AlCl_3_ group (*p* < 0.01). Meanwhile, no significant change was demonstrated between the control group and the groups with AzA resorption (Fig. 7C).

The results also demosterated a significant induction of CHOP gene expression in the AlCl_3_ group when compared to the control group [*F* (3, 28) = 12.68, *p* < 0.001]. Despite the reduction that AzA induced in the expression of this gene in the AzA+AlCl_3_ group, it was considerably more than the control group (*p* < 0.01) and statistically not very different than neither the AlCl_3_ group nor the AzA group (Fig. 7D).

### AChE activity in the hippocampus

**Figure 8.**
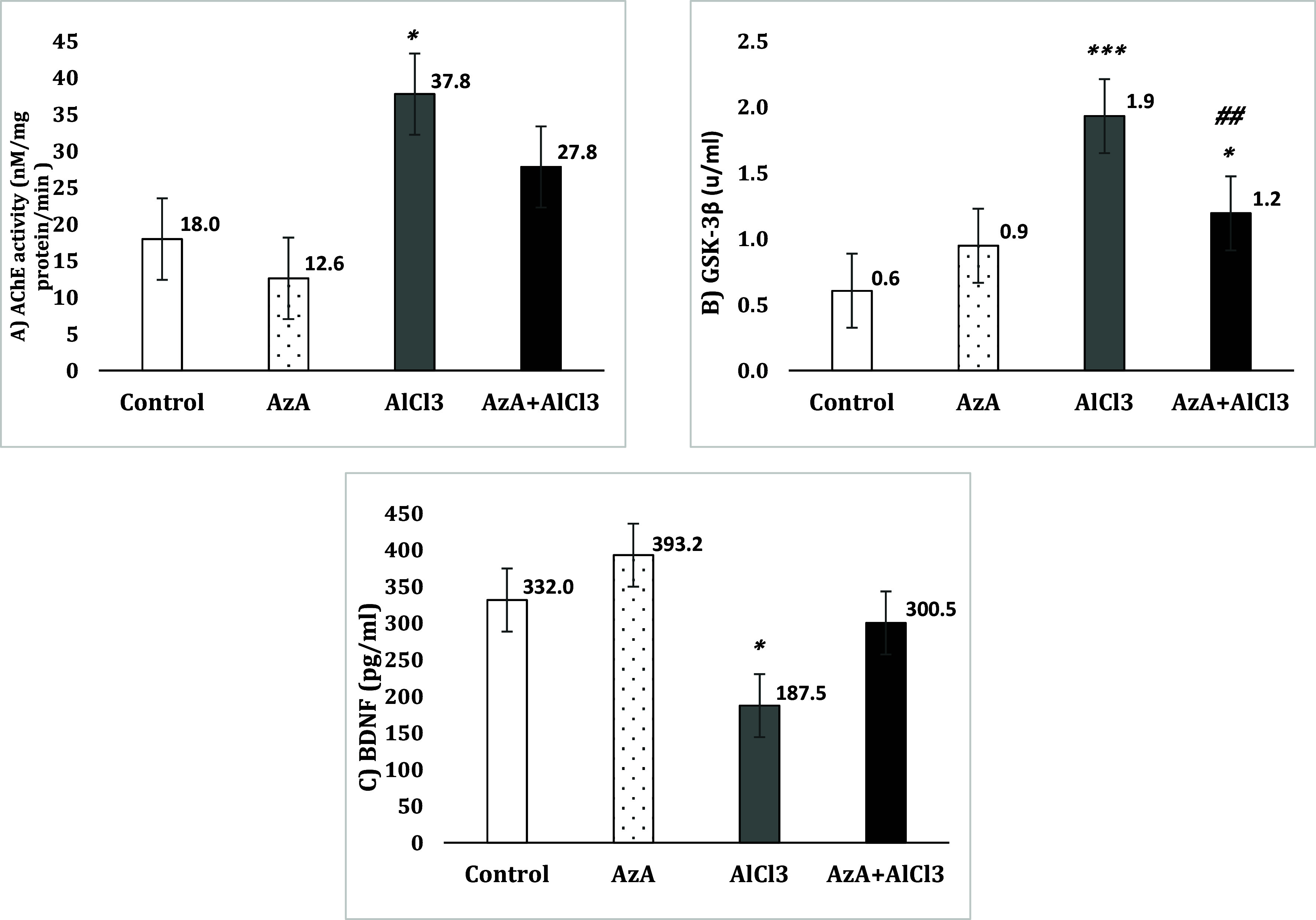
Effect of azelaic acid on AlCl_3_-induced changes of acetylcholinesterase activity, glycogen synthase kinase-3beta and brain derived neurotrophic factor in the hippocampus. The results were represented as mean±SEM (one-way ANOVA). (*) *p* < 0.05 and (***) *p* < 0.001 compared with the control group, (##) *p* < 0.01 compared with the AlCl_3_ group. AzA: azelaic acid, AChE: acetylcholinesterase, GSK-3β: glycogen synthase kinase-3beta, BDNF: brain-derived neurotrophic factor.

The evaluation of hippocampus samples showed noticeably boosted AChE activity in the AlCl-administerd group compared to the control group [*F* (3, 28) = 6.14, *p* < 0.05]. Although AzA decreased the effect of AlCl_3_ in the AzA+AlCl_3_ group and even reduced AChE activity in the AzA group in comparison to the control, both changes were not significant (Fig. 8A).

### GSK-3β activity in the hippocampus

Signal transduction assay reaction detected considerably increased activity of GSK-3β in the AlCl_3_ group in comparison to the control group [*F* (3, 28) = 15.11, *p* < 0.001] and the AzA group. Also, AzA significantly decreased the GSK-3β activity in the AzA+AlCl_3_ group compared to the AlCl_3_ group (*p* < 0.01), the enzyme activity level in this group was not notably different than the AzA group unlike the control group (*p* < 0.05) (Fig. 8B).

### BDNF levels in the hippocampus

The BDNF content of AlCl_3_ group was considerably decreased compared to the control group [*F* (3, 28) = 10.12, *p* < 0.05] and the AzA group, even though this decrement was not significant compared to AzA+AlCl_3_ group. However, despite the increased level of BDNF, the AzA group exhibited no significant change compared to the control group and the AzA+AlCl_3_ group (Fig. 8C).

## Discussion

Cognition is described as brain processes such as learning, memory, and executive functioning. It is operated by the frontal lobe, hippocampus, and other structures of the CNS (Harvey, 2019). Impairment of cognitive function takes place following damage to neuronal tissue and leads to the disruption of the individual’s performances in society and personal life. The excessive production of ROS and RNS, as well as inflammatory mediators in the CNS, damage to the cell signalling pathways particularly PI3K/Akt/mTOR and synapses are among the main causes of cognitive deficits (Das *et al*., 2023, Gutierrez and Limon, 2022).

Present study investigated protective effects of AzA against AlCl_3_-induced behavioural and cognitive deficits in male Wistar rats. AlCl_3_ could impair the NOR, MWM learning and memory, passive avoidance task, and cause significant provocation of anxiety-like behaviours in the EPM and OFM tests. Administration of the AlCl_3_ also, causes reduction in BDNF levels of hippocampus and intensification of AChE activity, GSK-3β, oxidative stress and neuroinflammatory biomarkers including protein carbonyl, MDA, TNF-α, IL-1, CHOP and NF-κB.

Al can simply cross the BBB via specific receptors with high affinity for transferrin, and accumulate in all the regions of the brain such as hippocampus (Exley, 2016, Skalny *et al*., 2021). Persistent Al exposure is followed by the reduction of dopamine level and purkinje cells that cause cholinergic disorder (Singla and Dhawan, 2017). Meantime, this metal can inhibit catalase, glutathione and glutathione S-transferase, increase nitrite and provoke the AChE activity leading to excessive reduction of ACh in the brain (Abu-Taweel and Al-Mutary, 2021, Khan *et al*., 2013). On the other hand, AlCl_3_ significantly increases the concentration of proteins such as Aβ_1-42_ (Zhang *et al*., 2022). Al accumulation also forces mitochondria to release cytochrome *c* and eventually the extreme production of ROS and RNS resulting in the activation of innate immune system of the brain (Abbas *et al*., 2022, Maksoud *et al*., 2020, Arab-Nozari *et al*., 2019). The immune system mediates the release of factors such as TNF-α, IL-1, CHOP and NF-κB and the degeneration of the neuronal cells. In agreement with the results of the previous researches, AlCl_3_-intoxicated rats in our study showed significantly higher AChE activity, inflammatory and oxidative stress factors in the hippocampus compared to the control group.

BDNF has been known to have an essential role in the regulation of synaptic plasticity and cognitive processes so that higher levels of BDNF slows down the rate of cognitive decline. Reduced BDNF levels are frequently associated with neurodegenerative conditions, such as Alzheimer’s disease, where they correlate with cognitive deficits. AlCl_3_ exposure has been shown to decrease BDNF expression in the brain, contributing to cognitive impairments observed in animal models. Our findings confirm that AlCl_3_ significantly reduces BDNF levels in the hippocampus, consistent with previous studies (Kasbe *et al*., 2015, Abbas *et al*., 2022, Zhang *et al*., 2022).

In addition to oxidative stress and neuroinflammation, Al would cause neuronal cell death through other mechanisms such as the activation of PI3K/Akt/mTOR signalling pathway (Skalny *et al*., 2021, Shang *et al*., 2020). PI3K/Akt/mTOR stimulates the expression of pro-apoptotic factors such as GSK-3β. GSK-3β is a key regulator in several cellular processes, including inflammation, apoptosis, and neuronal plasticity. Its dysregulation, often characterised by hyperactivity, is implicated in the pathogenesis of neurodegenerative diseases such as Alzheimer’s disease. AlCl_3_ exposure is known to increase GSK-3β activity, leading to the enhanced phosphorylation, decrease in glycogen concentration and ultimately apoptosis and neurodegeneration (Hamdan *et al*., 2022). In this study, we observed a significant increase in GSK-3β activity in the hippocampus following AlCl_3_ exposure, which aligns with previous findings that link GSK-3β hyperactivity to neurodegenerative processes.

The explained changes in the mentioned biochemical processes supported behavioural deficits of AlCl_3_-exposed animals indicated by memory impairments (Kasbe *et al*., 2015, Al-Amin *et al*., 2016) and anxiety-like behaviours (Singla and Dhawan, 2017, Abu-Taweel and Al-Mutary, 2021). In the EPM test, the administration of AlCl_3_ decreased the number of entries to the open arm, the time spent in the open arm, and locomotor activity. In the OFM test, AlCl_3_ administration decreased grooming number, distance moved, number of entries to the inner zone of the OFM chamber, and time spent in that area and additionally, it increased the rearing number of the rats. These results collectively indicate an exacerbation of anxiety in the AlCl_3_ group.

The findings from the passive avoidance test revealed that the AlCl_3_ group exhibited significantly less STL, spent more time in the DC, and had more entries to the DC compared to the control group. Furthermore, in both the learning and testing sessions of the MWM test, the AlCl_3_ group performed significantly worse than the control group. Additionally, their discrimination ratio in the NOR test was significantly decreased compared to the control group. The findings of these tests are evidence of the damaging effects of AlCl_3_ on the brain and cognitive functioning.

Using antioxidants is one of the strategies to prevent or treat oxidative stress-related diseases (Lee *et al*., 2020). AzA has been proved to have anti-inflammatory and antioxidant effects (Sieber and Hegel, 2014, Sauer *et al*., 2023). There is a report indicating neuroprotective effects of AzA against SH-SYSY-JNK3 cell apoptosis (Gan *et al*., 2011). This compound also reduced neurodegeneration in animal models of Parkinson’s disease (Sharmaa *et al*., 2021). Meanwhile, recent investigations suggested the possible inhibitory effect of AzA on PI3K/Akt/mTOR Signaling Pathway (Li *et al*., 2022). Based on these evidences, the effect of this organic compound on AlCl_3_ stimulated cognitive dysfunction was evaluated through animal behaviour and memory tests and biochemical assessment of hippocampus.

We observed that, in comparison to the AlCl_3_ group, the presence of AzA in the AzA+AlCl_3_ group led to significant improvements in various behavioural tests. AzA+AlCl_3_ group showed enhanced performance, specifically, in the NOR test, passive avoidance test, and MWM test. These results indicate that AzA may effectively counteract the learning and memory deficits induced by Al, as demonstrated in the AzA+AlCl_3_-treated rats.

The AzA+AlCl_3_ group exhibited significantly lower levels of anxiety compared to the AlCl_3_ group. This was evidenced by their increased entries to the open arm, extended time spent in the open arm, and higher locomotor activity in the EPM test. Additionally, this group displayed a higher grooming number and covered a longer distance in the OFM test. Furthermore, in the OFM test, there was an increase in the number of entries to the centre zone and time spent there, while the rearing number decreased following AzA+AlCl_3_ administration, when compared to the AlCl_3_ group.

AzA also could bring back the oxidation-reduction balance, as indicated by a significant decrease in TBARS and protein carbonyl content in the AzA+AlCl_3_ group compared to the AlCl_3_ group. These findings suggest that the neuroprotective effects of AzA may involve its role in managing oxidative stress and influencing downstream signalling networks like the PI3K/Akt pathway. By potentially inhibiting this pathway, AzA could reduce the activity of GSK-3β pro-apoptotic factor in the hippocampus of AzA+AlCl_3_ group compared to the rats only treated with AlCl_3_, thereby protecting against neuronal cell death. AzA+AlCl_3_ group had lower hippocampal inflammatory factors including TNF-α, IL-1β and NF-κB in comparison to the AlCl_3_ group. However, the decrease in the CHOP levels in the AzA+AlCl_3_ group was NOT noticeable when compared to the AlCl_3_-administerd group. Based on the statistics of the AzA+AlCl_3_ group, it appears that AzA was not able to effectively suppress the additive effect of AlCl_3_ on the AChE activity. However, AzA did manage to decrease the AChE activity of the AzA+AlCl_3_ group to a level where there was no significant difference observed when compared to the control group and the AzA group either. The non-significant change in AChE activity suggests that while AzA can counteract some of the neurotoxic effects of AlCl_3_, it may not be sufficient to completely reverse cholinergic dysfunction at the dose used in this study. This finding raises the possibility that higher doses of AzA or combination therapies may be required to achieve more robust neuroprotection.

Behavioural improvements in the learning and memory tests are likely underpinned by the observed molecular changes, such as the reduction in GSK-3β activity and the partial restoration of BDNF levels in the hippocampus of AzA+AlCl_3_-treated rats in comparison to the AlCl_3_ group. The increase in BDNF, although NOT statistically significant, may still contribute to the enhanced synaptic plasticity and cognitive function observed in the AzA+AlCl_3_ group, as BDNF is a key regulator of learning and memory processes.

## Conclusion

Based on the data obtained from our research, AzA indeed could alleviate AlCl_3-_induced behavioural alterations in rats. AzA reduced most of the oxidative and inflammatory disturbance induced by AlCl_3_. Findings of this study highlight the potential effect of AzA against neurocognitive impairments, but the sufficiency of these effects remained a question we couldn’t confidently answer. Although AzA could ameliorate cognitive functioning and limit the oxidative stress and neuroinflammation caused by AlCl_3_, further research is required in the future.

## References

[ref1] Abbas F , Eladl MA , El-Sherbiny M , Abozied N , Nabil A , Mahmoud SM , Mokhtar HI , Zaitone SA and Ibrahim D (2022) Celastrol and thymoquinone alleviate aluminum chloride-induced neurotoxicity: behavioral psychomotor performance, neurotransmitter level, oxidative-inflammatory markers, and BDNF expression in rat brain. Biomedicine & Pharmacotherapy 151, 113072. 35576663 10.1016/j.biopha.2022.113072

[ref2] Abu-Taweel GM and Al-Mutary MG (2021) Pomegranate juice moderates anxiety- and depression-like behaviors in alCl(3)-treated male mice. Journal of Trace Elements in Medicine and Biology 68, 126842. 34418746 10.1016/j.jtemb.2021.126842

[ref3] Al-Amin MM , Reza HM , Saadi HM , Mahmud W , Ibrahim AA , Alam MM , Kabir N , Saifullah AR , Tropa ST and Quddus AH (2016) Astaxanthin ameliorates aluminum chloride-induced spatial memory impairment and neuronal oxidative stress in mice. European Journal of Pharmacology 777, 60–69. 26927754 10.1016/j.ejphar.2016.02.062

[ref4] Alivand L , Younesi S , Tabrizian S , Kahnamouie-Aghdam F , Zeynizadeh S , Mazani M , Amani F and Mostafalou S (2024) Clinical presentations of PCOS in association with insulin resistance and serum esterase enzymes: a case-control study. Medicine & Health 19, 412–424.

[ref5] Arab-Nozari M , Zamani E , Latifi A and Shaki F (2019) Mitochondrial toxicity of aluminium nanoparticles in comparison to its ionic form on isolated rat brain mitochondria. Bratislavske lekarske listy 120, 516–522. 31602987 10.4149/BLL_2019_083

[ref6] Brodziak A , Kołat E and Różyk-Myrta A (2014) In search of memory tests equivalent for experiments on animals and humans. Medical Science Monitor 20, 2733–2739. 25524993 10.12659/MSM.891056 PMC4280055

[ref7] Butterfield DA and Boyd‐Kimball D (2019) Redox proteomics and amyloid β-peptide: insights into Alzheimer disease. Journal of Neurochemistry 151, 459–487. 30216447 10.1111/jnc.14589 PMC6417976

[ref8] Colomina MT and Peris- Sampedro F (2017) Aluminum and Alzheimer’s disease. Adv Neurobiol 18, 183–197. 28889268 10.1007/978-3-319-60189-2_9

[ref9] Das TK , Ganesh BP and Fatima-Shad K (2023) Common signaling pathways involved in alzheimer’s disease and stroke: two faces of the same coin. Journal of Alzheimer’s disease reports 7, 381–398. 10.3233/ADR-220108 PMC10200243 37220617

[ref10] Exley C (2016) The toxicity of aluminium in humans. Morphologie 100, 51–55. 26922890 10.1016/j.morpho.2015.12.003

[ref11] Gan M , Lin S , Zhang Y , Zi J , Song W , Hu J , Chen N , Wang L , Wang X and Shi J (2011) [Liposoluble constituents from Iodes cirrhosa and their neuroprotective and potassium channel-blocking activity]. Zhongguo Zhong Yao Za Zhi 36, 1183–1189. 21842646

[ref12] Golitabari N , Mohammadian F , Salari A-A and Amani M (2022) Neonatal NMDA blockade alters the LTP, LTD and cognitive functions in male and female wistar rats. Neuropharmacology 205, 108896. 34822815 10.1016/j.neuropharm.2021.108896

[ref13] Gutierrez BA and Limon A (2022) Synaptic disruption by soluble oligomers in patients with alzheimer’s and parkinson’s disease. Biomedicines 10, 1743–1758. 35885050 10.3390/biomedicines10071743 PMC9313353

[ref14] Hamdan AME , Alharthi FHJ , Alanazi AH , El-Emam SZ , Zaghlool SS , Metwally K , Albalawi SA , Abdu YS , Mansour RE , Salem HA , Abd Elmageed ZY and Abu-Elfotuh K (2022) Neuroprotective effects of phytochemicals against aluminum chloride-induced alzheimer’s disease through ApoE4/LRP1, Wnt3/β-catenin/GSK3β, and TLR4/NLRP3 pathways with physical and mental activities in a rat model. Pharmaceuticals (Basel) 15, 1008–1030. 36015156 10.3390/ph15081008 PMC9416484

[ref15] Harvey PD (2019) Domains of cognition and their assessment Dialogues clin neurosci, 21:227–237. 10.31887/DCNS.2019.21.3/pharvey PMC6829170 31749647

[ref17] Igbokwe IO , Igwenagu E , Igbokwe NA (2019) Aluminium toxicosis: a review of toxic actions and effects. Interdisciplinary toxicology 12, 45–70. 32206026 10.2478/intox-2019-0007 PMC7071840

[ref18] Kasbe P , Jangra A , Lahkar M (2015) Mangiferin ameliorates aluminium chloride-induced cognitive dysfunction via alleviation of hippocampal oxido-nitrosative stress, proinflammatory cytokines and acetylcholinesterase level. Journal of Trace Elements in Medicine and Biology 31, 107–112. 26004900 10.1016/j.jtemb.2015.04.002

[ref19] Khan KA , Kumar N , Nayak PG , Nampoothiri M , Shenoy RR , Krishnadas N , Rao CM and Mugdal J (2013) Impact of caffeic acid on aluminium chloride-induced dementia in rats. Journal of Pharmacy and Pharmacology 65, 1745–1752. 24236984 10.1111/jphp.12126

[ref20] Lee KH , Cha M and Lee BH (2020) Neuroprotective effect of antioxidants in the brain. International Journal of Molecular Sciences 21, 7152–7180. 32998277 10.3390/ijms21197152 PMC7582347

[ref21] Li L , Lu H , Zhang Y , Li Q , Shi S and Liu Y (2022) Effect of azelaic acid on psoriasis progression investigated based on phosphatidylinositol 3-kinase (PI3K)/Protein kinase B (AKT) signaling pathway. Clinical, Cosmetic and Investigational Dermatology 15, 2523–2534. 36447569 10.2147/CCID.S389760 PMC9701457

[ref22] Livingston G , Huntley J , Sommerlad A , Ames D , Ballard C , Banerjee S , Brayne C , Burns A , Cohen-Mansfield J , Cooper C , Costafreda SG , Dias A , Fox N , Gitlin LN , Howard R , Kales HC , Kivimäki M , Larson EB , Ogunniyi A , Orgeta V , Ritchie K , Rockwood K , Sampson EL , Samus Q , Schneider LS , Selbæk G , Teri L and Mukadam N (2020) Dementia prevention, intervention, and care: 2020 report of the lancet commission. Lancet 396, 413–446. 32738937 10.1016/S0140-6736(20)30367-6 PMC7392084

[ref23] Mahdavi P , Mokhtari S , Iranparvar M , Amani F , Mazani M and Mostafalou S (2024) Link of serum esterase enzymes with cognitive impairment in diabetic patients. Medicine & Health 19, 161–172.

[ref24] Maksoud HAA , Said AM , Abdeldaiem MA and Hassan MA (2020) Aluminum chloride induced inflammatory process in rat’s brain. Scholars International Journal of Biochemistry 3, 1–4.

[ref25] McDonald WM (2017) Overview of neurocognitive disorders. Focus (American Psychiatric Publishing) 15, 4–12. 31975834 10.1176/appi.focus.20160030 PMC6519631

[ref26] Mostafalou S , Baeeri M , Bahadar H , Soltany-Rezaee-Rad M , Gholami M and Abdollahi M (2015) Molecular mechanisms involved in lead induced disruption of hepatic and pancreatic glucose metabolism. Environmental Toxicology and Pharmacology 39, 16–26. 25434758 10.1016/j.etap.2014.11.001

[ref27] Mousavi-Nasab K , Amani M and Mostafalou S (2024) The effect of trientine on Alcl3-induced cognitive dysfunction and biochemical changes in the hippocampus of rats. Drug Research 74, 405–414. 39173674 10.1055/a-2381-6882

[ref28] PubChem [Internet] (2004) National Center for Biotechnology Information PubChem Compound Summary for CID 2266, Azelaic Acid. Bethesda, MD: National Library of Medicine (US). https://pubchem.ncbi.nlm.nih.gov/compound/Azelaic-Acid

[ref30] Saadati H , Esmaeili-Mahani S , Esmaeilpour K , Nazeri M , Mazhari S and Sheibani V (2015) Exercise improves learning and memory impairments in sleep deprived female rats. Physiology & Behavior 138, 285–291. 25447468 10.1016/j.physbeh.2014.10.006

[ref31] Sadegzadeh F , Sakhaie N , Dehghany R , Adak O and Saadati H (2020) Effects of adolescent administration of fluoxetine on novel object recognition memory, anxiety-like behaviors, and hippocampal brain-derived neurotrophic factor level. Life Science Part 1 Physiology & Pharmacology 260, 118338. 10.1016/j.lfs.2020.118338 32841662

[ref32] Sauer N , Oślizło M , Brzostek M , Wolska J , Lubaszka K and Karłowicz-Bodalska K (2023) The multiple uses of azelaic acid in dermatology: mechanism of action, preparations, and potential therapeutic applications. Postepy dermatologii i alergologii 40, 716–724. 38282869 10.5114/ada.2023.133955 PMC10809820

[ref33] Shang N , Zhang P , Wang S , Chen J , Fan R , Chen J , Huang T , Wang Y , Duncan J , Zhang L , Niu Q and Zhang Q (2020) Aluminum-induced cognitive impairment and PI3K/Akt/mTOR signaling pathway involvement in occupational aluminum workers. Neurotoxicity Research 38, 344–358. 32506341 10.1007/s12640-020-00230-z

[ref34] Sharmaa N , Khuranaa N , Muthuramanb A and Utrejac P (2021) Azelaic acid attenuatesrotenone-induced behavioural alterations in parkinson’s disease rat model. Plant Archives 21, 2333–2337.

[ref35] Sieber MA and Hegel JK (2014) Azelaic acid: properties and mode of action. Skin pharmacology and physiology 27, 9–17. 10.1159/000354888 24280644

[ref36] Singla N and Dhawan DK (2017) Zinc improves cognitive and neuronal dysfunction during aluminium-induced neurodegeneration. Molecular Neurobiology 54, 406–422. 26742519 10.1007/s12035-015-9653-9

[ref37] Skalny AV , Aschner M , Jiang Y , Gluhcheva YG , Tizabi Y , Lobinski R and Tinkov AA (2021) Molecular mechanisms of aluminum neurotoxicity: update on adverse effects and therapeutic strategies. Advances in neurotoxicology 5, 1–34. 34263089 10.1016/bs.ant.2020.12.001 PMC8276946

[ref38] Teleanu DM , Niculescu AG , Lungu II , RADU CI , Vladâcenco O , Roza E , Costăchescu B , grumezescu AM and Teleanu RI (2022) An overview of oxidative stress, neuroinflammation, and neurodegenerative diseases. International Journal of Molecular Sciences 23, 5938–5960. 35682615 10.3390/ijms23115938 PMC9180653

[ref39] Wang L (2018) Entry and deposit of aluminum in the brain. Advances in experimental medicine and biology 1091, 39–51. 30315448 10.1007/978-981-13-1370-7_3

[ref40] Zhang Z , Wu H , Qi S , Tang Y , Qin C , Liu R , Zhang J , Cao Y and Gao X (2022) 5-methyltetrahydrofolate alleviates memory impairment in a rat model of alzheimer’s disease induced by D-galactose and aluminum chloride. International journal of environmental research and public health 19, 16426–16440. 36554305 10.3390/ijerph192416426 PMC9779170

